# Gadofluorine M-enhanced MRI shows involvement of circumventricular organs in neuroinflammation

**DOI:** 10.1186/1742-2094-7-70

**Published:** 2010-10-18

**Authors:** Eva Wuerfel, Carmen Infante-Duarte, Robert Glumm, Jens T Wuerfel

**Affiliations:** 1 Experimental and Clinical Research Center, Charité - University Medicine Berlin, Charitéplatz 1, 10117 Berlin, Germany; 2 Department of Pediatrics, University Luebeck, Ratzeburger Allee 160, 23538 Luebeck, Germany; 3 Institute of Neuroradiology, University Luebeck, Ratzeburger Allee 160, 23538 Luebeck, Germany

## Abstract

**Background:**

Circumventricular organs (CVO) are cerebral areas with incomplete endothelial blood-brain barrier (BBB) and therefore regarded as "gates to the brain". During inflammation, they may exert an active role in determining immune cell recruitment into the brain.

**Methods:**

In a longitudinal study we investigated *in vivo *alterations of CVO during neuroinflammation, applying Gadofluorine M- (Gf) enhanced magnetic resonance imaging (MRI) in experimental autoimmune encephalomyelitis, an animal model of multiple sclerosis. SJL/J mice were monitored by Gadopentate dimeglumine- (Gd-DTPA) and Gf-enhanced MRI after adoptive transfer of proteolipid-protein-specific T cells. Mean Gf intensity ratios were calculated individually for different CVO and correlated to the clinical disease course. Subsequently, the tissue distribution of fluorescence-labeled Gf as well as the extent of cellular inflammation was assessed in corresponding histological slices.

**Results:**

We could show that the Gf signal intensity of the choroid plexus, the subfornicular organ and the area postrema increased significantly during experimental autoimmune encephalomyelitis, correlating with (1) disease severity and (2) the delay of disease onset after immunization. For the choroid plexus, the extent of Gf enhancement served as a diagnostic criterion to distinguish between diseased and healthy control mice with a sensitivity of 89% and a specificity of 80%. Furthermore, Gf improved the detection of lesions, being particularly sensitive to optic neuritis. In correlated histological slices, Gf initially accumulated in the extracellular matrix surrounding inflammatory foci and was subsequently incorporated by macrophages/microglia.

**Conclusion:**

Gf-enhanced MRI provides a novel highly sensitive technique to study cerebral BBB alterations. We demonstrate for the first time *in vivo *the involvement of CVO during the development of neuroinflammation.

## Background

The central nervous system (CNS) may no longer be considered immune privileged but rather a site of selective immune activity [1,2]. This so-called restricted immunity is warranted by the barrier function of capillary endothelium, which channels the entry of serum proteins and immune cells from the blood to the CNS or the cerebrospinal fluid (CSF), respectively [1]. Although the blood-brain barrier (BBB) covers most parts of the CNS, certain brain regions including the choroid plexus as well as structures that line the cavity of the third and of the fourth ventricle are devoid of a tight BBB and are in permanent contact to blood-born molecules and cells. These "exposed" areas, called circumventricular organs (CVO), are characterized by a dense capillary network with wide perivascular areas. Assumably, specialized ependymal cells, the tanycytes, act as a flexible barrier controlling the exchange of substances between CVO and the surrounding brain parenchyma as well as the CSF [3,4]. Besides neuroendocrine functions, CVO provide an access route for immune cells into the brain and might therefore guide CNS immune surveillance. The capacity of the choroid plexus to build a bridge for immune cells trafficking from the blood circulation into the CSF and the subarachnoid space was demonstrated in physiological [5] as well as under inflammatory conditions [6]. Immune activation was also reported in other CVO during experimental autoimmune encephalomyelitis (EAE), an animal model of multiple sclerosis (MS), indicating, that these unprotected CNS areas play a key role for pathological immune processes of the brain [7]. However, until recently no reliable method had been available to survey CVO *in vivo*. Assuming a crucial function as "gates to the brain" for immune cells, the visualization of alterations in CVO might become of diagnostic and therapeutic value for the assessment of neuroinflammatory conditions, similar to the detection of BBB impairment by gadopentetate dimeglumine- (Gd-DTPA) enhanced MRI, the current gold standard for the evaluation of disease activity in MS. New developments in high field strength MRI and novel contrast media provide optimized means to detect lesions and localize alterations in BBB integrity, even in small rodent disease models such as murine EAE [8]. The novel gadolinium-based contrast agent Gadofluorine M (Gf) was recently shown to facilitate the visualization of CNS lesions and cranial nerve inflammation [8-10]. Gf was originally applied for the detection of malignant lymph nodes [11], atherosclerotic plaques [12], or peripheral nerve damage [13].

In this study, we demonstrated for the first time that Gf-enhanced MRI represents a unique tool to *in vivo *visualize alterations of CVO. The magnitude of Gf accumulation in the choroid plexus and other CVO increased during active inflammation, correlated with disease activity, and could be used to differentiate between EAE animals and controls. Furthermore, Gf facilitated the detection of otherwise occult inflammatory CNS lesions.

## Methods

### Adoptive-transfer EAE

All experiments were approved by the local animal welfare committee and conformed to the European Communities Council Directive (86/609/EEC). For adoptive transfer EAE, female naïve SJL/J mice were immunized with an emulsion containing 250 μg PLP (murine proteolipid peptide p139-151; purity >95%, Pepceuticals, Leicester, UK) in equal volumes of phosphate buffered saline (PBS) and Complete Freunds Adjuvant (CFA, Difco Laboratories, Detroit, MI, USA) and 4 mg/ml Mycobacterium tuberculosis H37Ra (Difco Laboratories, Detroit, MI, USA) [14]. Ten days after immunization cells were extracted from draining lymph nodes and restimulated with 12.5 μg PLP/ml in cell culture medium (RPMI 1640 supplemented with 2 mM L-glutamine, 100 U/ml penicillin, 100 μg/ml streptomycin and 10% fetal calf serum) for 4 days at 37°C. Then 8-12 × 10^6 ^T-cell blasts in 100 μl PBS were injected intraperitoneally into syngenic recipients [15].

Mice were daily weighed and scored for neurological deficits as previously described [16]: 0, unaffected; 1, tail weakness or impaired righting on attempt to roll over; 2, paraparesis; 3, paraplegia; 4, paraplegia with forelimb weakness or complete paralysis; score >4, to be sacrificed. Mice with a score of 4 received an intraperitoneal injection of 200 μl glucose 5% daily.

### MRI analysis

After induction of EAE, mice underwent cerebral MRI between day 5 and 16 post T cell transfer on a 7 Tesla rodent scanner (Pharmascan 70/16AS, Bruker BioSpin, Ettlingen, Germany), applying a 20 mm RF-Quadrature-Volume head coil. Animals received anesthesia via facemask with 1.5 - 2.0% isoflurane (Forene, Abbot, Wiesbaden, Germany) delivered in 100% O_2 _under constant ventilation and body temperature control (Bio Trig System, Bruker BioSpin, Ettlingen, Germany).

We acquired axial and coronal T1-weighted images (MSME; TE 10.5 ms, TR 322 ms, 0.5 mm slice thickness, matrix 256 × 256, field of view (FOV) 2.8 cm) before and after intravenous (i.v.) injection of 0.2 mmol/kg bodyweight Gd-DTPA (gadopentetate dimeglumine, Magnevist, Bayer-Schering AG, Berlin, Germany), or 0.1 mmol/kg bodyweight Gf (Gadofluorine M, kindly provided by Drs. M. Reinhardt and B. Misselwitz, Bayer-Schering AG, Berlin, Germany). Gd-DTPA is the current gold standard for the detection of BBB breakdown in the CNS, showing as hyperintensity on T1-weighted images instantly after i.v. application. With a blood half-life of 20 min it is largely excreted from the organism after 3 h [17]. Gf is a modified amphiphilic gadolinium complex with a molecular weight of about 1.53 g/mol, also generating bright enhancement on T1-weighted images. Gf was designed by adding a perfluoroctyl chain to a gadolinium containing macrocycle. Gf interacts with hydrophobic proteins of the extracellular matrix such as collagen, proteoglycans, decorin and tenascin, and it is largely bound to serum albumin after i.v. administration [18]. It has a plasma half-life of 15.6 h [19]. The dose applied in this study was approved by previous MRI studies [8,9]; it is far below the estimated lethal dose of 5 mmol/kg [11].

We investigated a total number of 28 mice by MRI, comprising 21 EAE and 7 control mice. Fifteen EAE mice initially received Gd-DTPA. If BBB breakdown could be detected, Gf was applied and MRI repeated after 24 h, as established previously [8]. A subgroup of 4 EAE mice was additionally imaged to assess early kinetics 1 h and 6 h after Gf injection. Two mice were followed up until complete clinical remission in order to investigate longitudinal Gf signal decay. A further group of 6 EAE mice received Gf pre-labelled with the red fluorescent marker Cy3.5 (Gf-Cy3.5) for subsequent immunofluorescence histology. Seven naïve mice served as healthy controls. The kinetic of disease was exclusively studied *in vivo *owing to ethical concerns, which forced us to minimize the number of animals investigated. Histological analyses were performed after the final MR acquisitions.

### Histology

Mice were perfused for post mortem analysis in the acute disease phase within 2 to 5 days after onset. Following the final MRI, brain and spinal cord were prepared for histology, as previously described [8]. Every second slice was stained with hematoxylin and eosin (H&E) to assess inflammation. For the evaluation of Gf distribution, consecutive slices were stained with Hoechst 33258 nuclear stain (1:10000, Molecular Probes, Leiden, the Netherlands) to visualize cellular organization. We performed immunohistochemical staining against IBA-1 to identify macrophages/microglia, using the primary antibody rabbit anti-IBA-1 (1:1000, Wako Chemicals, Neuss, Germany) and goat anti-rabbit Cy2 (Amersham, Muenchen, Germany) as secondary antibody [8]. Selected sections were examined by epifluorescence microscopy and digitally photographed (Olympus BX-51, Hamburg, Germany).

### Data analysis

MRI data were coregistered and corrected for magnetic field inhomogeneity using MIPAV 6.1 (Center for Information Technology, National Institutes of Health, Bethesda, MD, USA). Statistical analysis was performed with GraphPad Prism 4.0c (GraphPad Software, Inc., San Diego, CA, USA). We determined the T1 lesion load of each individual EAE animal by calculating the volume of cranial Gf enhancement in T1-weighted sequences, applying a semi-automated procedure described previously [20]. In contrast to Gf, enhancement after Gd-DTPA administration was diffuse without clearly obtainable borders and furthermore, was often masked by strong intraventricular Gd-DTPA signal due to Gd-DTPA leakage into the CSF. Accordingly, we were not able to reliably quantify the volume of Gd-DTPA-enhancing lesions. The Gf lesion volume was correlated to 1) the EAE score at the day of imaging and 2) the day of clinical EAE onset after immunization, applying Spearman's nonparametric analysis.

For the assessment of Gf enhancement in CVO, regions of interest (ROI) were placed in corresponding positions in all animals, and the mean ROI signal intensity as well as the standard deviation was calculated. We evaluated the subfornicular organ (SFO), the organum vasculosum of the lamina terminalis (OVLT), the median eminence (ME) and the area postrema (AP) in coronal slices. The most reliable ROI placement of the choroid plexus could be achieved in axial slices of the lateral ventricles. Choroid plexus examination was generally more rater demanding due to its widespread expansion inside the ventricles with high interindividual anatomic shape differences, and was more prone to partial volume contaminations compared to other areas. The mean signal intensitiy of each ROI was expressed as Gf mean intensity ratio by division with the mean signal intensity of a masseter muscle ROI for unbiased and stable comparison between individuals.

Two-tailed Mann-Whitney-U tests were applied to express differences of the Gf mean intensity ratio in CVO between naïve versus EAE animals. Correlation analyses were performed between the Gf mean intensity ratio and 1) the EAE score at the day of MRI and 2) the day of clinical disease onset using Spearman's nonparametric analysis. A receiver-operating characteristic (ROC) was used to analyze the validity of the Gf mean intensity ratio in CVO as diagnostic test to distinguish between EAE mice and controls.

## Results

### Clinical EAE course and contrast-enhancing lesions (CEL)

Transfer of PLP-specific T cells into recipient animals resulted in the development of clinical signs in 18 out of 21 mice within 6-13 (mean 9.6) days after immunization. However, contrast-enhancing lesions (CEL) were detectable in all 21 mice in T1-weighted images after Gd-DTPA and Gf application. Animals progressed to a peak EAE score of 2.6 (1-4) within 2 days after onset, and developed an average Gf-enhanced T1-hyperintense lesion volume of 8.31 mm^3 ^(table 1). The Gf lesion volume correlated significantly with the EAE score at imaging, but not with the day of clinical EAE onset after immunization (table 2).

**Table 1:** Clinical EAE course and Gf lesion load

Mouse	EAE score at Gf imaging	Gf lesion load in mm^3^	Day of clinical onset
1	1	30.5933	10
2	3	22.0243	9
3	3	19.7933	11
4	1	17.3736	13
5	4	13.7473	6
6	2	12.2391	7
7	1	10.4559	9
8	2	9.6347	9
9	1	7.4567	9
10	2	6.3080	9
11	2	6.0699	9
12	2	4.8076	11
13	1	3.2377	11
14	1	3.0483	11
15	1	1.8621	11
16	0	1.4209	11
17	0	1.3857	-
18	3	0.9528	8
19	1	0.8987	9
20	0	0.7135	-
21	0	0.5301	-

mean (SD)	1.48 (+/-1.12)	8.31 (+/-8.34)	9.61 (+/-1.69)

After detection of cranial blood-brain barrier breakdown by Gd-DTPA, 0.1 mmol/kg bodyweight Gf was applied, and MRI repeated after 24 h. The EAE score at the day of Gf imaging and the corresponding lesion volume in T1-weighted Gf-enhanced images are presented. The day of clinical onset is given as days after T cell transfer.

**Table 2:** Gf enhancement correlates with clinical EAE parameters

	EAE score at Gf imaging			Day of clinical onset		
	Gf lesion volume	SFO	AP	Gf lesion volume	SFO	AP
Spearman r	0.56	0.66	0.53	-0.09	-0.45	-0.35
95% CI	0.15 to 0.80	0.30 to 0.85	0.08 to 0.79	-0.54 to 0.41	-0.74 to -0.01	-0.70 to 0.14
P value	0.009	0.001	0.021	0.732	0.043	0.147
Significance	**	**	*	no	*	no

Correlation of clinical EAE parameters to total Gf lesion volume in T1-weighted images and Gf enhancement (mean intensity ratio) in the subfornicular organ (SFO) and the area postrema (AP). Correlations for other circumventricular organs were not statistically significant.

*: P value < 0.05; **: P value < 0.01; CI: confidence interval

**Figure 1:**
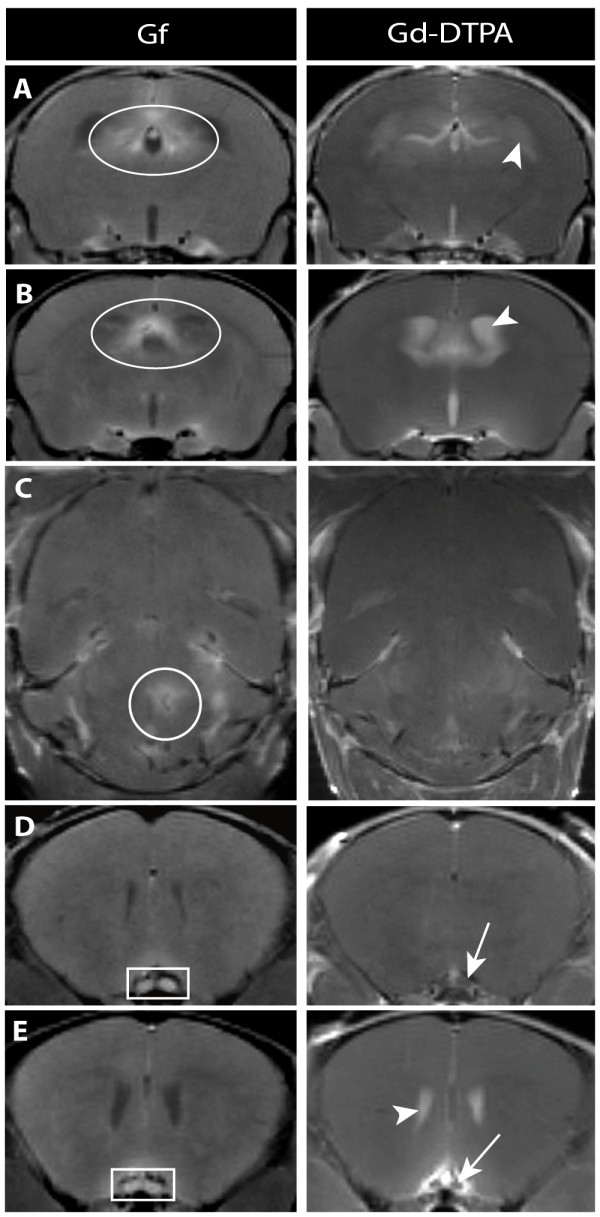
**Gf facilitates the detection of inflammatory plaques**. Coronal (A, B, D, E) and axial (C) T1- weighted images are depicted 24 h after Gf injection (left column) and immediately after Gd-DTPA injection (right column). Periventricular pathology was better assessable applying Gf (A and B: ovoid). The disruption of the blood-CSF barrier frequently caused leakage of contrast agent into the CSF (A, B, E: arrowhead), initially obscuring periventricular lesions. A parenchymal midbrain lesion is shown exemplary (C: encircled) that was not seen with Gd-DTPA. Visualization of optic neuritis (D and E: square) was markedly improved by Gf, since neighboring intravascular signal prohibited the unambiguous determination on Gd-DTPA-enhanced images (D and E: arrow).

Inflammatory plaques were widely distributed throughout the brain, with predominance to the brainstem and the periventricular region. In 15 mice receiving both, Gd-DTPA and Gf, a total number of 61 CEL was detected. Among these, 26 were exclusively visible after Gf administration, but not on Gd-DTPA-enhanced MRI. The visualization of optic nerve inflammation was particularly facilitated by Gf application (table 3). Gf-enhancing plaques appeared concise with well-delineated borders in comparison to more diffusely Gd-DTPA-enhancing lesions (figure 1).

**Table 3:** Contrast-enhancing lesions (CEL) exclusively detected by Gf

Brain region	Total number of CEL	Number of CEL detected with Gd-DTPA and Gf	Number of CEL detected exclusively with Gf	Percentage of CEL detected exclusively with Gf
Brain stem	14	10	4	28.6
Periventricular	11	9	2	18.2
Optic nerve	10	1	9	90
Trigeminal nerve	10	6	4	40
Cerebellum	9	6	3	33.3
Vestibular nerve	5	2	3	60
Cortex	2	1	1	50

	61	35	26	42.6

Distribution and number of contrast-enhancing lesions (CEL) after Gd-DTPA and Gf administration in 15 mice investigated with both contrast agents.

**Figure 2:**
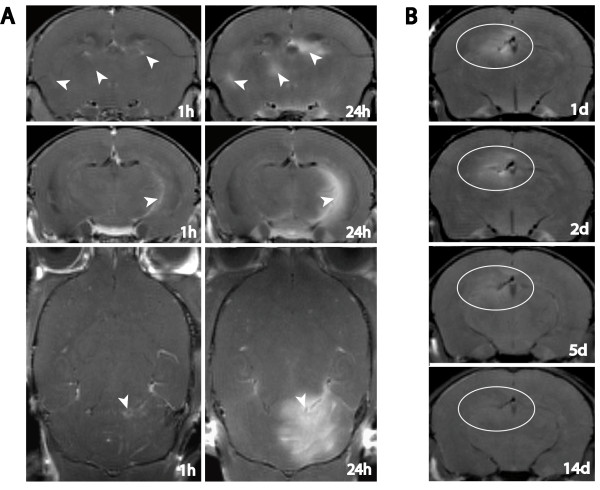
**Time course of Gf enhancement**. **A**: Inflammatory plaques located periventricularly (upper row), close to the parahippocampal fissure (middle row), and within the cerebellum (lower row) are depicted to exemplify the minor signal enhancement 1 h post Gf application compared to the vast parenchymal enrichment 24 h after Gf injection. **B**: Gradual Gf signal decline over a time period of 14 days is demonstrated in a periventricular lesion.

Gf tissue enhancement evolved with time delay compared to Gd-DTPA, which indicated BBB breakdown instantly after application. After 1 h, Gf CEL were detectable with circumscribed subtle parenchymal signal enhancement, and developed further within 24 h (figure 2A, arrowheads). T1-hyperintensity maximum was reached 24 h after application, followed by a gradual signal drop until complete remission to baseline values after 14 days (figure 2B).

### Visualization of CVO alterations by Gf-enhanced MRI

**Figure 3:**
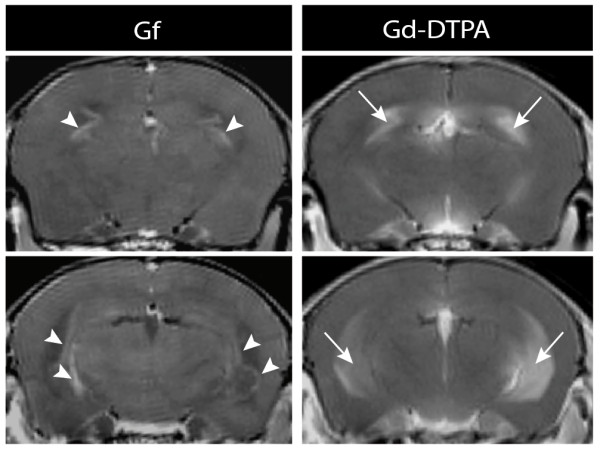
**Early Gf enhancement of the choroid plexus**. Coronal T1-weighted images are depicted 1 h after Gf injection (left column) and immediately after Gd-DTPA injection (right column). Applying Gf, distinct enhancement of the choroid plexus (arrow heads) and other CVO was evident early after intravenous injection and allowed for a reliable quantitative signal analysis. In contrast, after injection of Gd-DTPA, the disruption of the blood-CSF barrier frequently caused leakage of contrast agent into the CSF (arrows) obscuring the choroid plexus and periventricular lesions.

In addition to inflammatory plaques, Gf revealed alterations in CVO during acute CNS inflammation. Unlike the delayed accumulation in inflammatory plaques, bright enhancement of the choroid plexus was evident already 1 h after Gf injection. Applying Gd-DTPA, the evaluation of the choroid plexus was not reliably feasible, since it was frequently obscured by leakage of Gd-DTPA into the CSF (figure 3).

**Figure 4:**
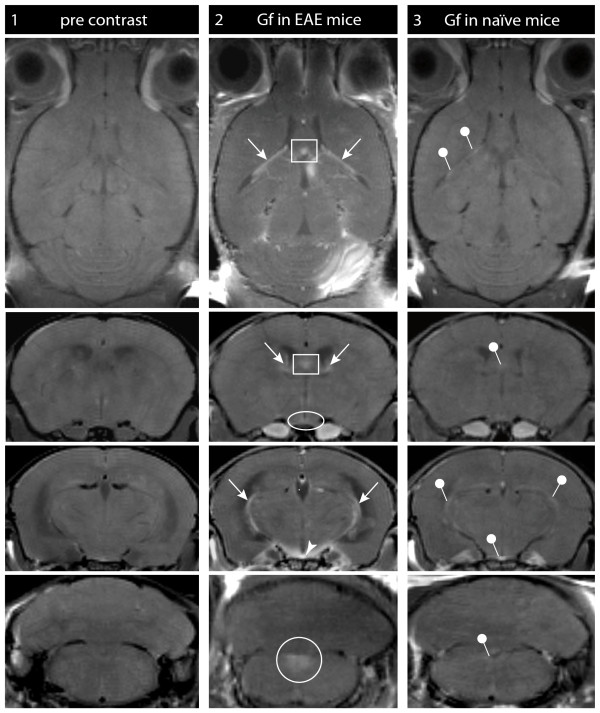
**Gf enhancement in circumventricular organs (CVO)**. Gf distinctly enhanced the choroid plexus and other CVO in naïve control mice and more pronounced after induction of EAE: T1-weighted images of the CVO are shown in axial (row 1) and coronal (row 2-4) orientation. Images were acquired prior to administration of contrast agent (column 1), and 24 h after Gf application in EAE (column 2) and naïve control mice (column 3). Enhancement of the choroid plexus (arrows), the subfornicular organ (square), the organum vasculosum of the lamina terminalis (ovoid), the median eminence (arrowhead) and the area postrema (circle) are clearly marked in the EAE mice (column 2). In naïve mice, only subtle CVO enhancement was detectable (column 3, pins).

Gf enhancement of the choroid plexus was a prominent finding in all 21 EAE mice investigated (figure 4, arrow). Gf accumulation was also observed in other CVO including the OVLT (figure 4, ellipse), the SFO (figure 4, square), the ME (figure 4, arrowhead) and the AP (figure 4, circle). Since CVO may represent major entry sites for immune cells into the CNS also under physiological conditions [6,21,22], we were interested to see whether Gf could visualize CVO "gates" in naïve animals, too. In all 7 healthy control mice, subtle Gf enhancement in CVO was detectable (figure 4, 3^rd ^column), although considerably less pronounced compared to EAE mice. We did not assess Gf enhancement in healthy mice longitudinally.

**Figure 5:**
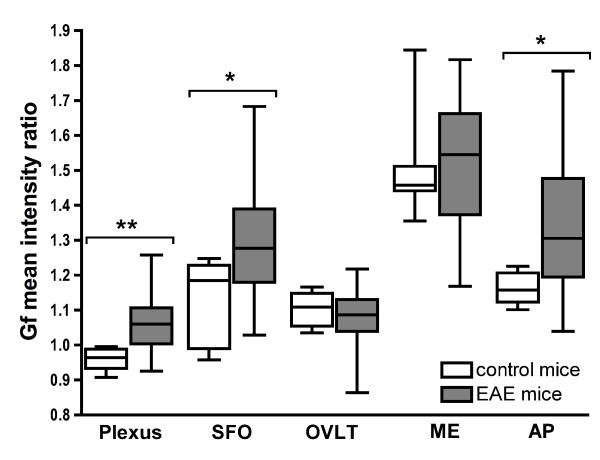
**Quantitative comparison of Gf enhancement in circumventricular organs (CVO)**. Gf enhancement was quantitatively assessed computing the Gf mean intensity ratio of the CVO in T1-weighted images 24 h after Gf application. The comparison between EAE and naïve control animals was performed using two-tailed Mann-Whitney-U tests. Gf enhancement in EAE mice was significantly higher in the choroid plexus (**: P = 0.008), the subfornicular organ (*: P = 0.020) and the area postrema (*: P = 0.016). SFO: subfornicular organ; OVLT: organum vasculosum of the lamina terminalis; ME: median eminence; AP: area postrema.

For a quantitative analysis of differences between EAE animals and naïve mice, we calculated the mean signal intensity ratio 24 h after Gf application in the CVO within these two groups (figure 5). The Gf mean intensity ratio was significantly higher in EAE mice compared to controls in the choroid plexus (p = 0.008), the SFO (p = 0.020) and the AP (p = 0.016). No difference was detected for OVLT and ME.

### Gf enhancement in CVO correlates with disease severity

**Figure 6:**
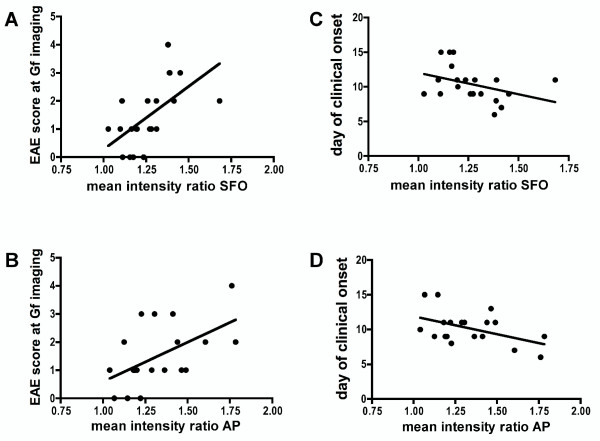
**Gf enhancement correlates with clinical parameters**. Correlation analyses were performed according to Spearman. The signal intensity (Gf mean intensity ratio) of the subfornicular organ (SFO) (A, P = 0.001) and the area postrema (AP) (B, P = 0.021) 24 h post injection correlated significantly to the corresponding EAE scores at the time of scanning. Additionally, the time needed from T cell transfer to disease onset correlated inversely with the mean intensity ratio of the SFO (C, P = 0.043) but not the AP (D, P = 0.147). Each dot depicts the data point of one mouse. For further statistics see table 2.

Within EAE animals, Gf mean intensity ratios of SFO and AP correlated closely with the EAE score at the day of the MRI investigation; mice with higher disease severity showed significantly brighter Gf enhancement (table 2 and figure 6A and 6B). Additionally, the Gf mean intensity ratio of the SFO correlated inversely with the time span until onset of clinical disease after T cell transfer; accordingly, Gf enhancement of SFO was significantly brighter in EAE mice with early disease onset (table 2 and figure 6C). The quantitative analysis of the choroid plexus was technically more demanding compared to CVO regions surrounded by brain parenchyma such as SFO and AP, due to its expansion throughout the ventricles with high interindividual anatomical shape differences. Correlations between clinical EAE features and choroid plexus enhancement failed to reach statistical significance, potentially due to partial volume contaminations of CSF when conducting a standardized ROI based semi-automated quantitative analysis.

### Gf enhancement of CVO differentiates EAE from control animals

We were further interested whether the quantitative assessment of Gf enhancement in different CVO could serve as diagnostic tool to distinguish between EAE and healthy mice. ROC curves were calculated separately for the Gf mean intensity ratio of the choroid plexus, the SFO and the AP. The area under the ROC curve was highly significant for the choroid plexus (p = 0.007) as well as for the SFO (p = 0.018) and the AP (p = 0.014). The Gf mean intensity ratio of the choroid plexus was the best discriminator to identify EAE animals with a sensitivity of 89% and a specificity of 80% using a cut-off value of 0.9826 (table 4).

**Table 4:** Gf enhancement differentiates between EAE animals and healthy controls

	Choroid plexus	SFO	AP
Area under the ROC curve	0.90	0.81	0.84
Standard error (95% CI)	0.07 (0.77 to 1.03)	0.09 (0.64 to 0.98)	0.08 (0.68 to 1.01)
P value (two-tailed)	0.007	0.018	0.014
Significance	**	*	*
Cutoff MIR	>0.9826	>1.232	>1.189
Sensitivity (95% CI)	89% (65 to 99%)	67% (41 to 87%)	82% (57 to 96%)
Specificity (95% CI)	80% (28 to 100%)	86% (42 to 100%)	83% (36 to 100%)
Likelihood ratio	4.44	4.67	4.94

ROC analysis of the Gf enhancement (mean intensity ratios) in circumventricular organs, differentiating between EAE animals and controls. Exemplary cut-off values and the resulting sensitivity, specificity and likelihood ratio are presented for each region

ROC: receiver operating characteristic; SFO: subfornicular organ; AP: area postrema; *: P value < 0.05; **: P value < 0.01; MIR: mean intensity ratio; CI: confidence interval

### Gf accumulates in the extracellular matrix and is incorporated by monocytic cells

**Figure 7:**
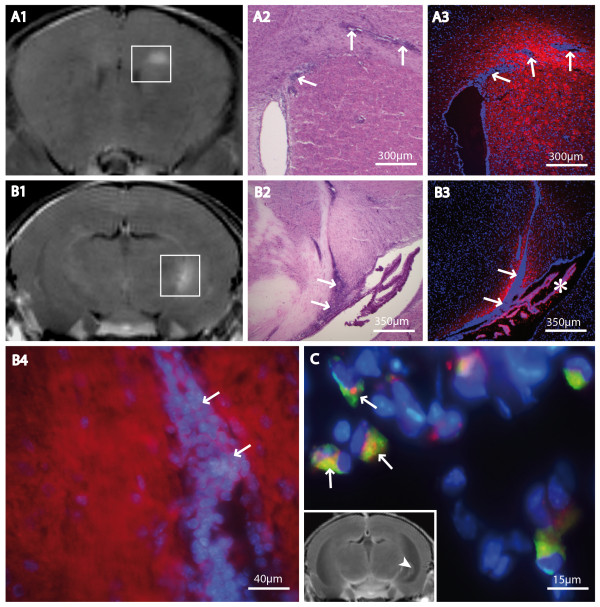
**Histological correlations**. The tissue distribution of fluorescence-labeled Gf was histologically assessed at two time points, 24 h (A and B) and 72 h (C) after intravenous Gf injection, and correlated to MRI. Coronal T1-weighted MR images 24 h after Gf injection are shown. Gf-enhancing lesions are marked by squares (A1 and B1). Two exemplary lesions studied after 24 h are shown, one with a periventricular inflammatory focus close to the corpus callosum (A) and a second spreading from the hippocampal fimbria to the choroid plexus (*) (B). Corresponding H&E stained (A2 and B2) and fluorescence microscopy (A3, B3, B4; red: Gf, blue: Hoechst 33258) slices depict typical inflammatory plaques, including cellular infiltrations (arrows), halo-like surrounded by Gf. A detail is given in higher magnification to point out the diffusely extracellular accumulation of Gf (B4). After 72 h, Gf uptake into macrophages/microglia was evident. In the choroid plexus, internalized Gf became visible in numerous IBA-1 positive cells (arrows in C; red: Gf; green: IBA-1; blue: Hoechst 33258). The MR image is shown to localize the detail (C: arrowhead).

In order to investigate cellular or molecular mechanisms of Gf tissue accumulation, we used carbocyanine dye (Cy3.5) prelabeled contrast agent. MR images were acquired 24 h and 72 h post application. Directly after MR measurements, mice were sacrificed and analyzed histologically. Twentyfour hours post injection, Gf-Cy3.5 produced a strong signal in immune fluorescence microscopy, matching the sites of contrast enhancement in MRI (figure 7A and 7B). Fluorescence was most intense within the center of the lesion and vanished towards the periphery (figure 7A3 and 7B3). Gf appeared to enrich homogeneously in the extracellular matrix in foci with high immune cell infiltration (figure 7B4). In corresponding H&E staining, these areas were identified as inflammatory perivascular cuffs typically seen in EAE (figure 7A2). Lesion sites were often found in vicinity to CVO. Please note the high number of periventricular lesions (table 1) in the proximity to the choroid plexus. Occasionally, the cell formation suggested migration of immune cells from CVO to inflammatory cuffs (figure 7A2). Gf enrichment was also detected in inflammatory sites located at the meninges (figure 7B). However, this was always associated with a focal inflammatory lesion; we did not detect a generalized meningeal Gf enhancement. Although the MR signal did not change significantly within the first 72 h, microscopically Gf formed scattered spots of bright red fluorescence neighbouring cell nuclei after initial homogeneous tissue fluorescence, likely due to the accumulation in cytosolic vesicles. Counterstaining with IBA-1 revealed an incorporation of Gf-C3.5 by macrophages/microglia, predominantly in regions with prominent cellular infiltrates detected by H&E staining. A particularly high density of IBA-1 positive cells with intracellular Gf-C3.5 deposits was detected in the choroid plexus (figure 7C).

## Discussion

This study provides the first *in vivo *evidence for the participation of the choroid plexus and other CVO in neuroinflammation. Applying the novel MRI contrast agent Gf in high field MRI, we successfully visualized alterations in the CVO, which in part, correlated to the severity of CNS inflammation. Transmigration across the choroid plexus is a well defined entry route for leucocytes into the CNS [1]. Already under physiological conditions, immune cells home to the choroid plexus and other CVO [23]. Macrophages and dendritic cells, key players in CNS antigen presentation, have been localized to the choroid plexus of naïve mice [6,24]. Recent histopathological studies stressed the role of the CVO also during inflammation, suggesting an initial recruitment of encephalitogenic T cells via the choroid plexus in EAE: Antigen presentation and subsequent reactivation of T lymphocytes is facilitated due to the presence of macrophages and microglia [25] and the upregulation of major histocompatibility complex antigens in these areas [7,26]. Expression of ICAM-1 and VCAM-1, molecules that are crucial for lymphocyte adhesion and transmigration, were induced on choroid plexus epithelium [6] and other CVO during inflammation [7].

Although MRI has emerged as a powerful tool to assess disease activity in MS and EAE, alterations of CVO during inflammation have not been monitored *in vivo *up to date. Nevertheless, the invasion of macrophages into inflammatory plaques was demonstrated *in vivo *in a recent study on MS patients. This finding was partially independent from simultaneous BBB breakdown, and the route of entry is still not completely understood [27]. We hypothesized that Gf-enhanced MRI might help to elucidate kinetics of CNS immune surveillance. Gf was originally developed as a marker for detecting lymph node metastasis in MR lymphography [11]. Meding et al. demonstrated, that Gf binds to serum albumin and components of the extracellular matrix such as collagens, proteoglycans, fibronectin and tenascin [18], explaining a possible mechanism of Gf accumulation in atherosclerotic plaques [12,28]. Furthermore, Gf is taken up by macrophages *in vivo *and *in vitro *[13,29] and thus could highlight spots of immune activity in autoimmune neuritis, EAE and peripheral nerve degeneration [8,9,13,30]. Recently, Gf was also applied to detect disease progression in an animal model of muscular dystrophy [31].

Here we demonstrate, that Gf accumulates weakly in the choroid plexus and other CVO involved in gating immune cell migration into the CNS in naïve mice, indicating an immunological function of those organs under physiological conditions. Interestingly, after initialization of EAE, pronounced Gf enhancement of the choroid plexus, the SFO and the AP became visible. Whereas leakage of Gd-DTPA into the CSF often prevented a reliable evaluation of the choroid plexus or periventricular brain regions in EAE mice, Gf-enhanced MRI allowed for the detailed quantitative evaluation of signal alterations in these areas. A correlation analysis in EAE mice exposed that high Gf enhancement of the SFO and the AP went along with an early disease onset and severe clinical affection. Regarding the SFO, these associations were even higher than the correlation of the same clinical parameters to the Gf lesion volume. Furthermore, we found that calculating the Gf mean intensity ratio in CVO can be applied as diagnostic tool to discriminate EAE from control animals, with a sensitivity of 89% and a specificity of 80% for the choroid plexus. In histological sections, we could confirm an accumulation of Gf in the choroid plexus stroma initially and a subsequent internalization into macrophages/microglia resident in the choroid plexus after 72 h. However, we did not detect significant alterations of the enhancement pattern in OVLT and ME during EAE. The predominance of the observed changes in periventricular and brain stem CVO might reflect a heightened regional vulnerability in developing inflammatory plaques during EAE in these areas [8].

Furthermore, we noted a high sensitivity of Gf to detect BBB leakage and associated parenchymal inflammation, particularly optic neuritis, resulting in a 42% increased number of contrast-enhancing lesions compared to Gd-DTPA-enhanced MRI, in line with a recent report by Bendszus et al. [9]. In the circulation, Gf is largely bound to albumin [18]. Brain parenchymal enhancement is due to a locally disturbed BBB and subsequent capturing of Gf molecules by protein interaction [10]. There is no evidence for an active transport mechanism of Gf across the intact BBB. Gf accumulated with delay within CNS lesions yielding a signal intensity peak after 24 h in contrast to Gd-DTPA that immediately passed the disrupted BBB. Gf enhancement, as quantified in our study, thus reflects the accumulation during 24 h of focal BBB disruption. High binding affinity to plasma albumin and extracellular matrix proteins may explain the delayed initial CNS tissue accumulation but also the persisting presence of Gf within the brain parenchyma for up to 10 days [9,18]. The inflammatory cascade in EAE includes a plethora of effector mechanisms such as secretion of matrix metalloproteases and other digesting enzymes by immune cells [32] initiating tissue degradation. Proteins liberated during this process are likely to trap Gf once it passed the disrupted BBB [10]. The histological analysis applying fluorescent Gf likewise indicated a diffuse extracellular enrichment pattern. The clearance of Gf from the brain parenchyma after 14 days might be a result of a gradual loss of local molecule binding affinity as well as the general clearance and molecular turnover at lesion sites. We also noted a local uptake of Gf molecules by macrophages/microglia. However, we could not establish a correlation to repair mechanisms such as remyelination (data not shown).

Despite a delayed brain parenchymal enhancement Gf accumulated in CVO already 1 h after application. CVO are primarily exposed to Gf molecules due to the dense capillary network devoid of a tight BBB. Endothelial cells of the choroid plexus and other CVO express proteoglycans, fibronectin and other extracellular matrix proteins [33,34], which may bind Gf. During CNS inflammation, such molecules are upregulated and mediate adhesion and subsequent transmigration of immune cells into the CNS [33,35]. Thus, adhesion to extracellular matrix proteins presented on CVO endothelium might explain both, Gf enhancement in CVO of healthy control mice and increased enhancement under inflammatory conditions.

## Conclusions

In summary, in this first *in vivo *MRI investigation visualizing inflammatory alterations in the CVO, Gf enhancement correlated to disease severity as well as to time of onset, indicating an active participation of the CVO in CNS inflammation. Gf enhancement in CVO was a good discriminator between healthy and EAE animals. Our results suggest that Gf enhancement could serve as sensitive marker for different hallmarks of CNS inflammation, including molecular "arming", immune cell recruitment, BBB breakdown and tissue damage. However, extensive preclinical testing is needed before a clinical application of Gf. This is eligible since Gf could not only facilitate the paraclinical diagnosis and follow-up of neuroinflammatory diseases such as MS but may also help to elucidate basic principles of CNS immunity.

## Competing interests

The authors declare that they have no competing interests.

## Authors' contributions

EW carried out the animal experiments and histological procedures, performed the statistical analyses, participated in MR scanning and drafted the manuscript. CID participated in the design of the study and helped to draft the manuscript. RG helped with the histological stainings. JTW conceived and designed the study, performed MR scanning and drafted the manuscript. All authors read and approved the final manuscript.
